# Supplementary material for: A novel computational approach for predicting complex phenotypes in Drosophila (starvation-sensitive and sterile) by deriving their gene expression signatures from public data
Source: PLoS One. 2020 Oct 26;15(10):e0240824. doi: 10.1371/journal.pone.0240824 (PMC7588067; doi:10.1371/journal.pone.0240824)
Supplement: S1 File — (PDF) [file pone.0240824.s001.pdf]

Supporting Information for

A novel computational approach for predicting complex phenotypes in *Drosophila* (starvation-sensitive and sterile) by deriving their gene expression signatures from public data

Dobril K. Ivanov[1,2]*, Gerrit Bostelmann[1], Benoit Lan-Leung[2], Julie Williams[2], Linda Partridge[3,4,¶], Valentina Escott-Price[2,¶], Janet M. Thornton[1,¶]

[1]European Molecular Biology Laboratory, The European Bioinformatics Institute (EMBL-EBI), Wellcome Trust Genome Campus, Hinxton, Cambridge, UK
[2]UK Dementia Research Institute at Cardiff (UKDRI), Cardiff University, College of Biomedical and Life Sciences, Hadyn Ellis Building, Cardiff, UK
[3]Max Planck Institute for Biology of Ageing, Cologne, Germany
[4]Institute of Healthy Ageing, and Department of Genetics, Evolution and Environment, UCL, London, UK
* Corresponding author
E-mail: IvanovD1@cardiff.ac.uk (DKI)
[¶]These authors are joint senior authors of this work (LP, VEP and JMT are Joint Senior Authors)

Supplementary Materials and Methods

Normalising gene-expression values

Raw gene-expression data (cel files) were downloaded from the EBI's ArrayExpress (https://www.ebi.ac.uk/arrayexpress/). Separately, for each experiment the raw data were summarised and normalised by using the Robust Multichip Average (*rma* function without background normalisation, part of bioconductor's package *affy* [1]. Summarised probe-sets were mapped to transcripts using bioconductor's package *drosophila2.db*. Transcripts not mapping to any known or predicted genes were excluded from further analysis. Log2-normalised expression data for all experiments that exhibit a particular phenotype were combined in a single dataset.

Generation of the Molecular Signatures (Linear-mixed effects model)

To assess the statistical significance of each gene we used logistic regression (*glm* function in R). For the LMEM, we utilised the *lmer* function (bobyqa optimiser) within the *lme4* package, part of bioconductor. For the LMEM, we utilised the *lmer* function within the *lme4* package, part of bioconductor.

Gene Ontology (GO) enrichment analysis

The Wilcoxon rank sum test, as implemented in Catmap ([2]), was used to perform functional analysis to test for significant enrichment of Gene Ontology categories. FlyBase gene identifiers were mapped to Gene Ontology identifiers (FlyBase version FB2018_02) using custom programs.

Supplementary Figures and Tables

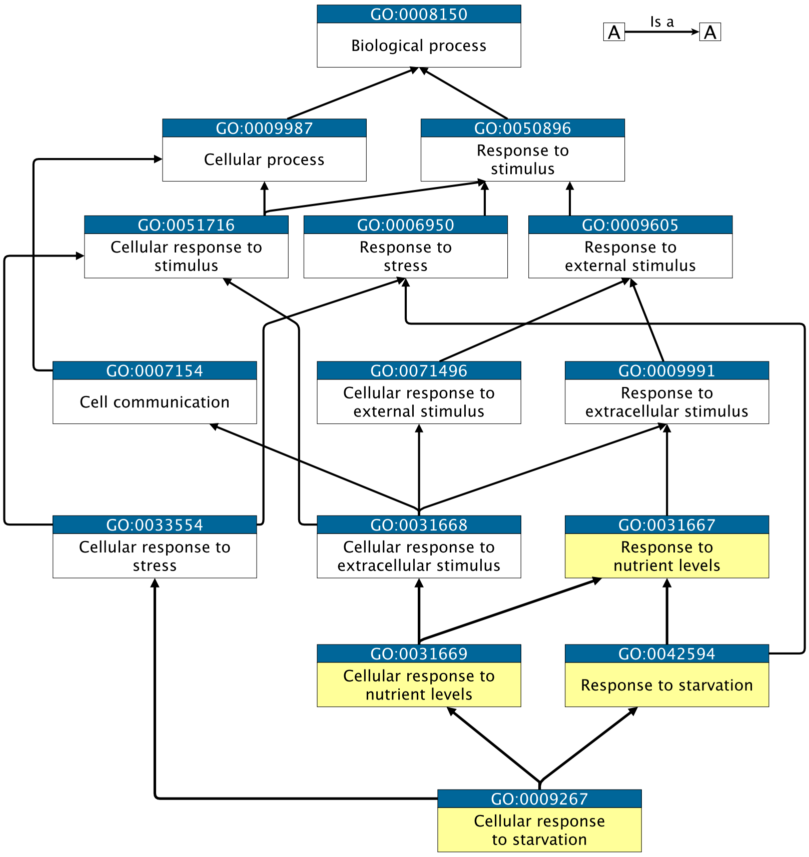

S1 Fig. Representative GO terms associated with the starvation sensitive phenotype. Boxes represent nodes and arrows represent edges. Nodes filled with yellow are the GO terms used to assess if the molecular signature is associated with the starvation sensitive phenotype. Data derived from https://www.ebi.ac.uk/QuickGO

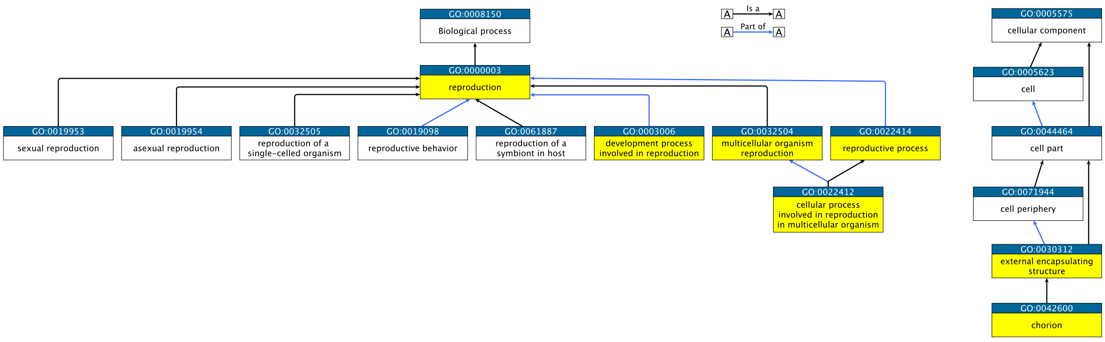

S2 Fig. Representative GO terms associated with the sterile phenotype. Boxes represent nodes and arrows represent edges. Nodes filled with yellow are the GO terms used to assess if the molecular signature is associated with the sterile phenotype. Data derived from https://www.ebi.ac.uk/QuickGO

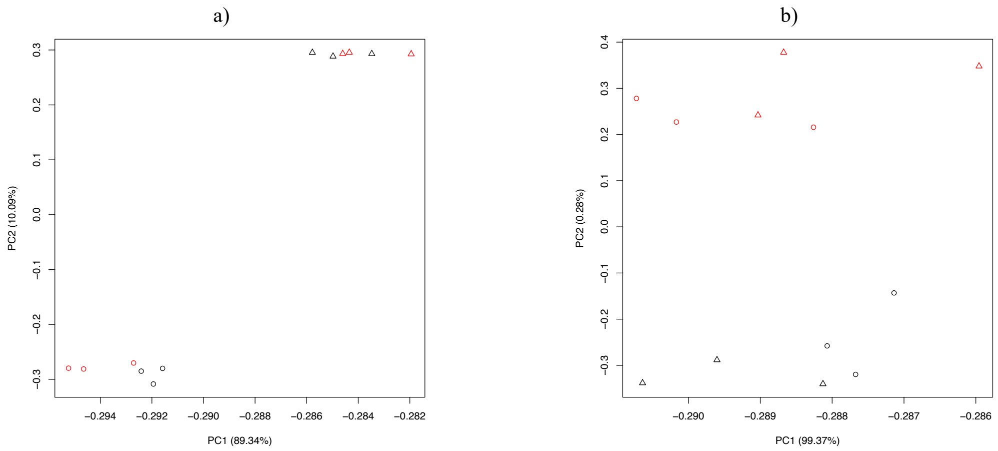

S3 Fig. PCA before and after batch effect correction for the *loj*. a) *loj* log-2 normalised values without batch effect correction b) *loj* log-2 normalised values after batch effect correction with *ber*; controls and mutants are labelled with black and red symbols respectively; circles and triangles represent samples derived from abdomen and head/thorax respectively

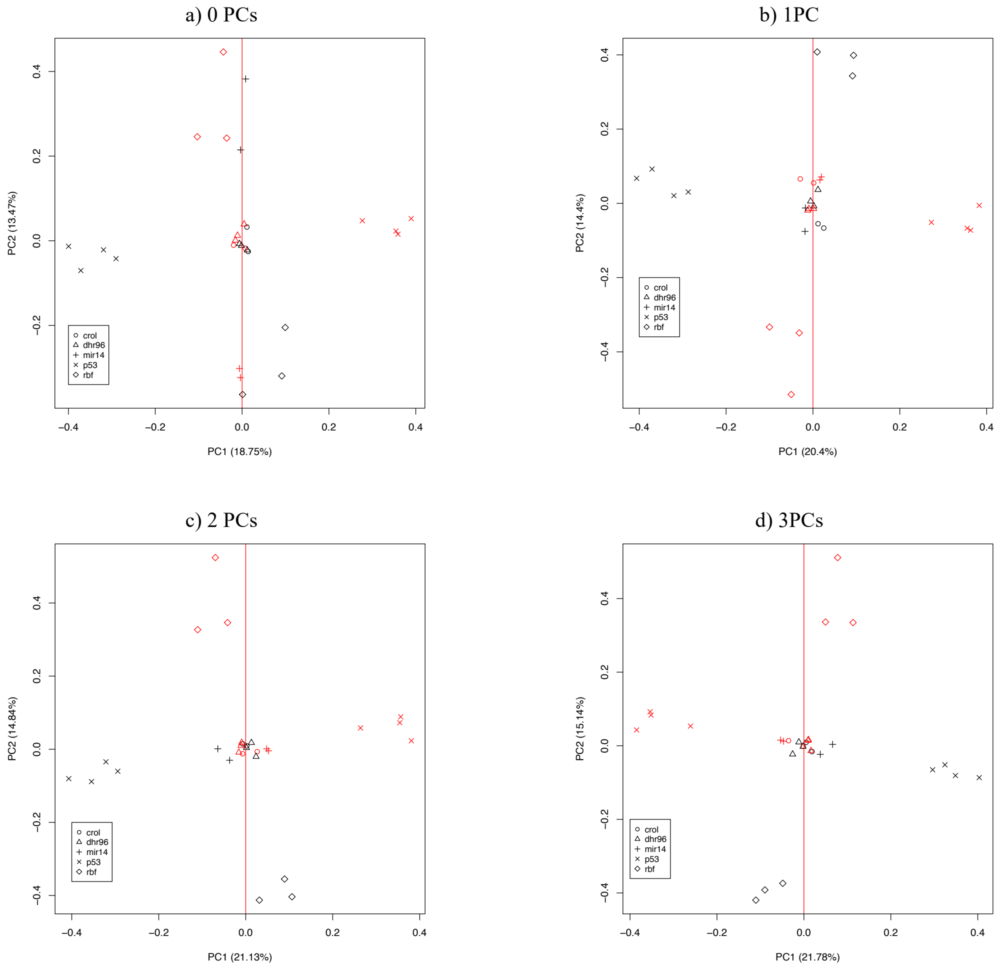

e) 4 PCs

f) 5 PCs

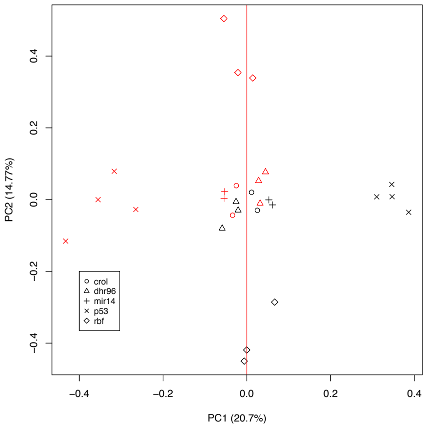

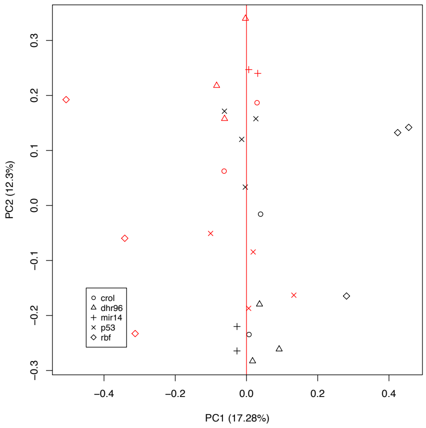

g) 6 PCs

h) 7 PCs

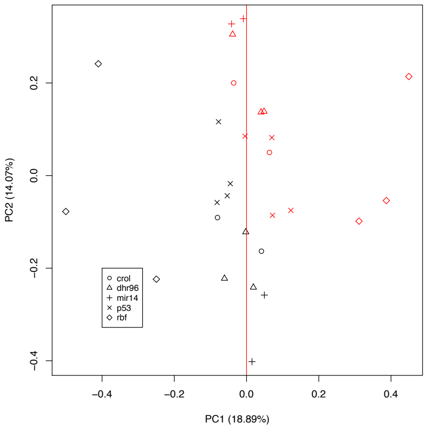

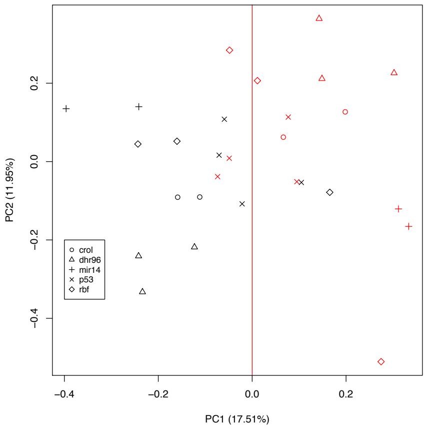

S4 Fig. PCA plots of LMEM with consecutive PCs of the starvation sensitive phenotype. a) LMEM with 0 PCs; b) LMEM with 1 PCs; c) LMEM with 2 PCs; d) LMEM with 3 PCs; e) LMEM with 4 PCs; f) LMEM with 5 PCs; g) LMEM with 6 PCs; h) LMEM with 7 PCs

a) 0 PCs

b) 1 PC

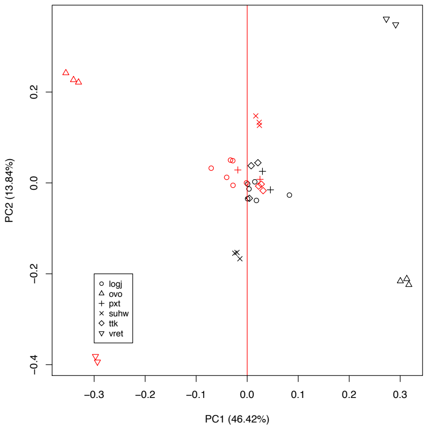

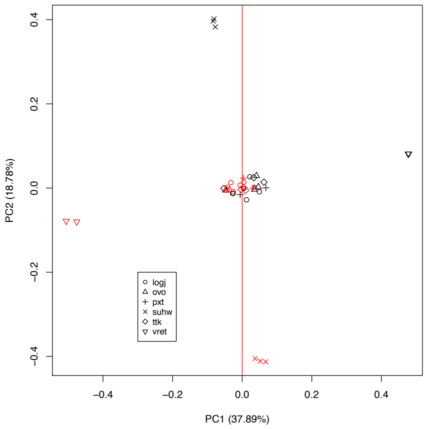

c) 2 PCs

d) 3 PCs

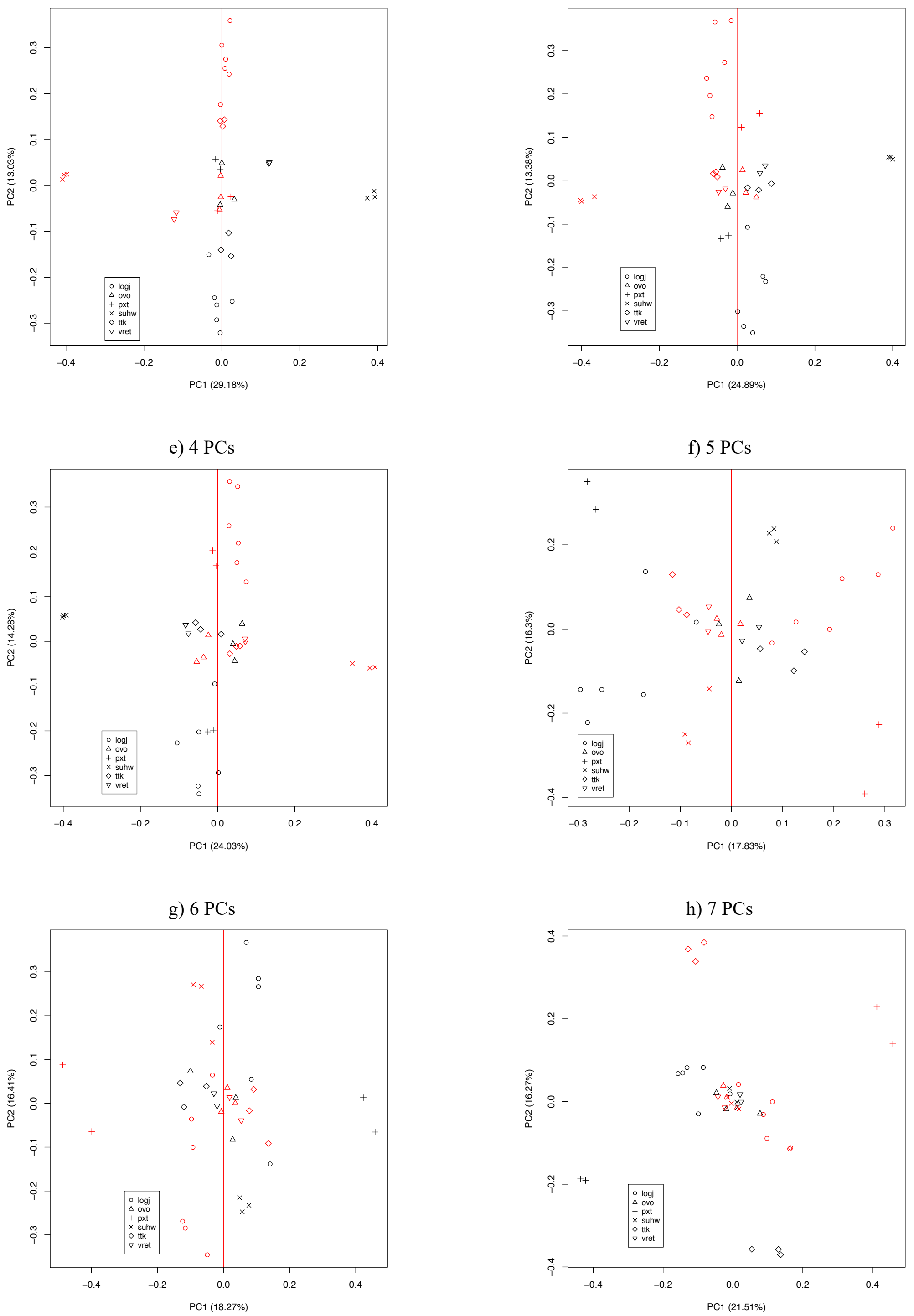

S5 Fig. PCA plots of LMEM with consecutive PCs of the sterile phenotype. a) LMEM with 0 PCs; b) LMEM with 1 PCs; c) LMEM with 2 PCs; d) LMEM with 3 PCs; e) LMEM with 4 PCs; f) LMEM with 5 PCs; g) LMEM with 6 PCs; h) LMEM with 7 PCs

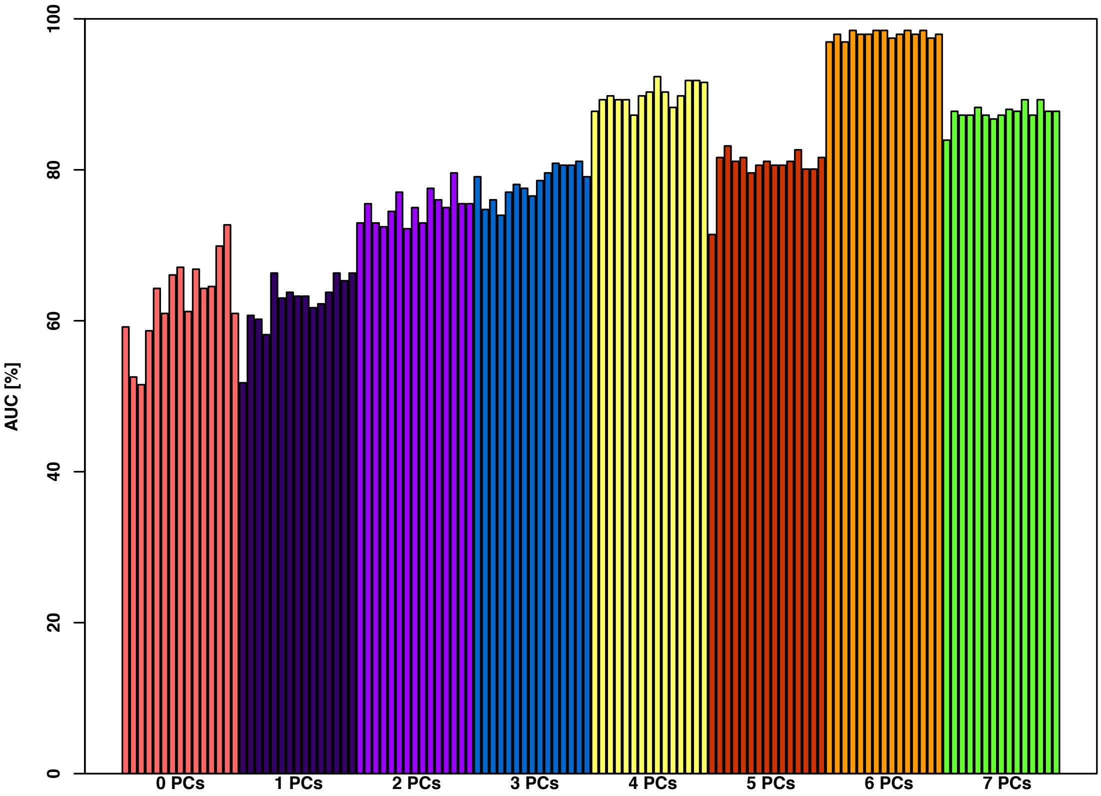

S6 Fig. AUC leave-one-out cross-validation using different number of top genes (starvation-sensitive phenotype)
AUC- Area Under the Curve; Each bar (from left to right) represents a one leave-one-out cross-validation using 50, 100, 200, 300, 400, 500, 600, 700, 800, 900, 1000, 1500, 2000, 2500 and 3000 genes within each of the PCs (represented by different colours)

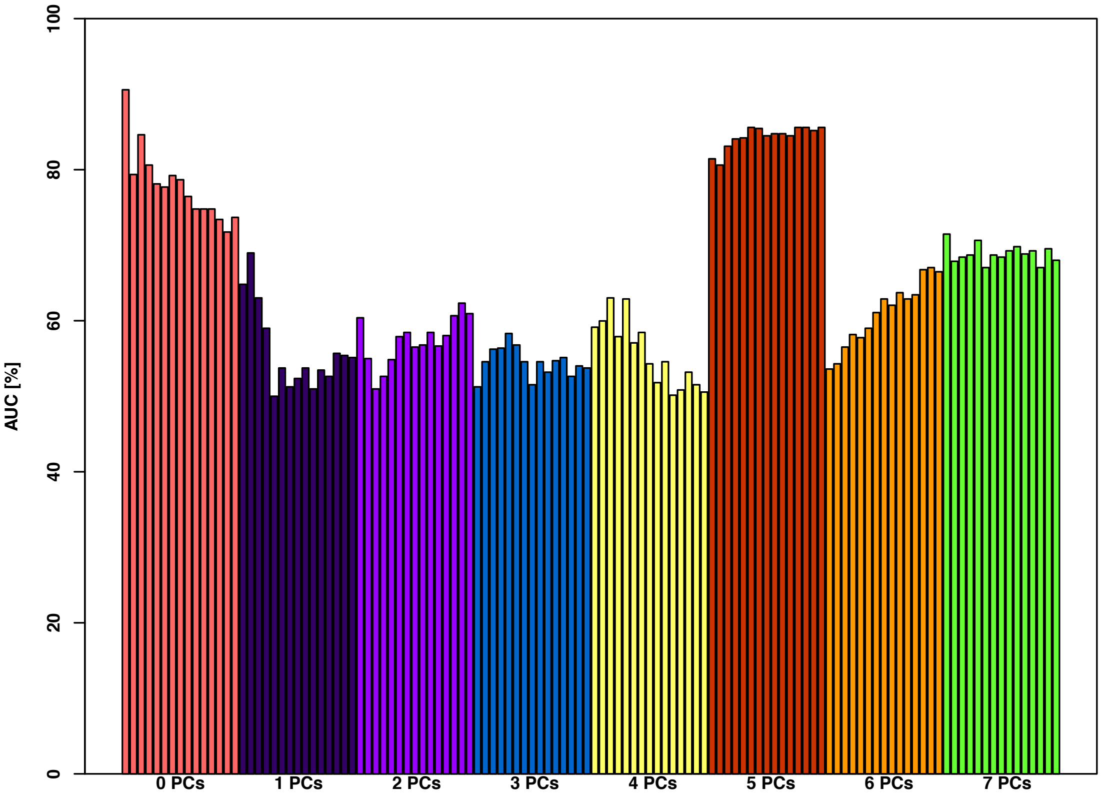

S7 Fig. Fig. AUC leave-one-out cross-validation using different number of top genes (sterile-sensitive phenotype)
AUC- Area Under the Curve; Each bar (from left to right) represents a one leave-one-out cross-validation using 50, 100, 200, 300, 400, 500, 600, 700, 800, 900, 1000, 1500, 2000, 2500 and 3000 genes within each of the PCs (represented by different colours)

Gene[a]	Accession ID[b]	N replicates		Age [days]	Tissue	Sex
		controls	mutants			
dhr96 (FBgn0015240; Hormone receptor-like in 96)	E-GEOD-18576 [3]	3	3	9	whole body	male
mir-14 (FBgn0262447; mir-14 stem loop)	E-GEOD-20202 [4]	2	2	5	head	male
rbf (FBgn0015799; Retinoblastoma-family protein)	E-GEOD-24978 [5]	3	3	0	3rd instar larvae	mixed
p53 (FBgn0039044)	E-GEOD-37404 [6]	4	4	0	3rd instar larvae	mixed
crol (FBgn0020309; crooked legs)	E-GEOD-8775 [7]	2	2	42	whole body	female

S1 Table Data used to derive the molecular signature for the starvation sensitive phenotype (FBcv:0000708). [a]FlyBase gene symbol with gene identifier and gene name in brackets; [b]EBI's ArrayExpress accession identifier with reference in brackets; all perturbed genes are knockouts

Gene[a]	Accession ID[b]	Number replicates		Age [days]	Tissue	Sex
		controls	mutants			
loj (FBgn0061492; logjam)	E-GEOD-10940 [8]	3	3	4	head/thorax	female
ovo (FBgn0003028)	E-GEOD-48145 [9]	3	3	15	whole body	female
pxt (FBgn0261987; Peroxinectin-like)	E-GEOD-29815 [10]	4	3	4	ovarian follicle	female
su(HW) (FBgn0003567; Suppressor of Hairy wing)	E-GEOD-36528 [11]	3	3	0	ovary	female
ttk (FBgn0003870; tramtrack)	E-GEOD-42758 [12]	3	3	1.5	ovary	female
vret (FBgn0263143; vreteno)	E-GEOD-30360 [13]	2	2	7	ovary	female

S2 Table Data used to derive the molecular signature for the sterile phenotype (FBcv:0000366). [a]FlyBase gene symbol with gene identifier and gene name in brackets; [b]EBI's ArrayExpress accession identifier with reference in brackets; all perturbed genes are knockouts

GO ID	GO name	*crol* FDR p-value	*dhr96* FDR p-value	*mir-14* FDR p-value	*p53* FDR p-value	*rbf* FDR p-value
GO:0009267	cellular response to starvation	**2.85E-08**	1.00E+00	6.11E-01	7.41E-02	7.80E-01
GO:0031669	cellular response to nutrient levels	**1.29E-08**	1.00E+00	5.74E-01	7.30E-02	7.93E-01
GO:0042594	response to starvation	**5.73E-05**	1.00E+00	3.46E-01	**3.54E-02**	3.68E-01
GO:0031667	response to nutrient levels	**1.33E-04**	1.00E+00	2.87E-01	**2.72E-02**	4.42E-01
GO:0031668	cellular response to extracellular stimulus	2.96E-08	1.00E+00	5.88E-01	5.05E-02	7.85E-01
GO:0033554	cellular response to stress	9.71E-11	1.00E+00	4.00E-01	1.73E-05	1.12E-07
GO:0009991	response to extracellular stimulus	1.90E-04	1.00E+00	3.01E-01	1.85E-02	4.34E-01
GO:0071496	cellular response to external stimulus	4.10E-07	1.00E+00	4.14E-01	2.70E-02	7.96E-01
GO:0007154	cell communication	3.85E-01	1.00E+00	9.77E-01	6.92E-02	2.67E-01
GO:0009605	response to external stimulus	3.74E-04	1.00E+00	2.07E-02	8.63E-03	1.64E-01
GO:0006950	response to stress	5.47E-11	1.00E+00	2.93E-02	3.69E-08	6.60E-08
GO:0051716	cellular response to stimulus	4.15E-09	1.00E+00	2.72E-01	1.18E-04	1.42E-05
GO:0050896	response to stimulus	4.47E-04	1.00E+00	9.94E-03	6.61E-03	1.85E-03
GO:0009987	cellular process	4.23E-11	1.00E+00	2.27E-04	2.17E-36	1.01E-18
GO:0008150	biological process	2.14E-01	1.00E+00	3.81E-01	1.19E-03	4.00E-02

S3 Table Individual experiment GO enrichment analysis (starvation sensitive phenotype). FDR p-value is the p-value corrected for multiple hypothesis testing using False Discovery Rate, accounting for all GO terms tested. GO terms directly related to starvation with FDR p-values < 0.05 are labelled in bold

GO ID	GO name	*loj* FDR p-value	*ovo* FDR p-value	*pxt* FDR p-value	*suhw* FDR p-value	*ttk* FDR p-value	*vret* FDR p-value
GO:0003006	developmental process involved in reproduction	**1.62E-03**	**7.25E-14**	**1.70E-29**	**2.49E-05**	**3.70E-08**	**1.39E-21**
GO:0022412	cellular process involved in reproduction in multicellular organism	**1.32E-04**	**6.40E-15**	**4.07E-32**	**1.65E-04**	**4.11E-09**	**3.22E-22**
GO:0022414	reproductive process	**3.38E-03**	**7.65E-12**	**1.13E-25**	**1.65E-04**	**1.01E-08**	**9.45E-19**
GO:0032504	multicellular organism reproduction	1.00E+00	1.00E+00	1.00E+00	1.00E+00	1.00E+00	1.00E+00
GO:0000003	reproduction	1.00E+00	1.00E+00	1.00E+00	1.00E+00	1.00E+00	1.00E+00
GO:0030312	external encapsulating structure	1.00E+00	**1.90E-05**	**9.23E-10**	**1.18E-16**	**1.68E-04**	**9.69E-31**
GO:0042600	chorion	1.00E+00	**3.15E-04**	**1.41E-10**	**1.82E-15**	**4.24E-05**	**1.21E-28**

S4 Table Individual experiment GO enrichment analysis (sterile phenotype). FDR p-value is the p-value corrected for multiple hypothesis testing using False Discovery Rate, accounting for all GO terms tested. FDR p-values < 0.05 are labelled in bold

**GO term**	**FDR p (0 cov)**	**FDR p (1 cov)**	**FDR p (2 cov)**	**FDR p (3 cov)**	**FDR p (4 cov)**	**FDR p (5 cov)**	**FDR p (6 cov)**	**FDR p (7 cov)**
BP GO:0009267 cellular response to starvation	1.86e-01	**4.13e-03**	**2.90e-03**	**2.37e-03**	**6.18e-03**	7.21e-02	1.65e-01	2.99e-01
BP GO:0031669 cellular response to nutrient levels	1.86e-01	**3.92e-03**	**2.39e-03**	**2.00e-03**	**5.01e-03**	5.97e-02	1.37e-01	2.63e-01
BP GO:0042594 response to starvation	5.59e-01	1.66e-01	1.36e-01	1.23e-01	1.72e-01	5.04e-01	5.29e-01	6.92e-01
BP GO:0031667 response to nutrient levels	6.70e-01	2.64e-01	2.43e-01	2.28e-01	2.65e-01	6.59e-01	6.40e-01	8.31e-01
AUC (200genes)	0.52	0.60	0.73	0.76	0.90	0.83	0.97	0.87

S5 Table Top GO terms for the starvation sensitive molecular signature. FDR p-value is the p-value corrected for multiple hypothesis testing using False Discovery Rate, accounting for all GO terms tested. Cov- number of Principal Components included in the linear-mixed effect model. BP- Biological Process; FDR p-values < 0.05 are labelled in bold; AUC- Area Under the Curve

**GO term**	**FDR p (0 cov)**	**FDR p (1 cov)**	**FDR p (2 cov)**	**FDR p (3 cov)**	**FDR p (4 cov)**	**FDR p (5 cov)**	**FDR p (6 cov)**	**FDR p (7 cov)**
BP GO:0003006 developmental process involved in reproduction	**2.46e-14**	**1.63e-03**	**9.16e-03**	**6.98e-03**	**3.18e-03**	**1.91e-02**	**1.06e-03**	5.25e-02
BP GO:0022412 cellular process involved in reproduction in multicellular organism	**3.05e-16**	**3.99e-04**	**2.78e-03**	**1.83e-03**	**7.14e-04**	**7.39e-03**	**5.46e-04**	**3.80e-02**
BP GO:0022414 reproductive process	**1.64e-12**	**1.37e-04**	**9.01e-04**	**1.56e-03**	**4.14e-04**	**1.91e-02**	**2.41e-03**	1.70e-01
BP GO:0032504 multicellular organism reproduction	1.00e+00	1.00e+00	1.00e+00	1.00e+00	1.00e+00	9.84e-01	9.97e-01	1.00e+00
BP GO:0000003 reproduction	1.00e+00	1.00e+00	9.99e-01	1.00e+00	1.00e+00	9.72e-01	9.93e-01	1.00e+00
CC GO:0030312 external encapsulating structure	**2.74e-05**	**3.14e-04**	3.06e-01	6.61e-01	9.20e-01	6.57e-01	5.86e-01	5.55e-01
CC GO:0042600 chorion	**1.45e-04**	**3.14e-04**	1.31e-01	4.56e-01	8.72e-01	6.76e-01	6.61e-01	6.18e-01
AUC (200genes)	0.85	0.63	0.51	0.56	0.63	0.83	0.57	0.58

S6 Table Top GO terms for the sterile molecular signature. FDR p-value is the p-value corrected for multiple hypothesis testing using False Discovery Rate, accounting for all GO terms tested. Cov- number of Principal Components included in the linear-mixed effect model. BP- Biological Process; CC- Cellular Component; FDR p-values < 0.05 are labelled in bold; AUC- Area Under the Curve

EBI Experiment ID	N con	N mut	EBI Control Experiment Factor Value	EBI Mutant Experiment Factor Value	µ Control Prob	µ Mutant Prob
E-GEOD-8775	2	2	female|wild type genotype	female|crol mutant	0.83	0.89
E-GEOD-18576	3	3	wild type genotype	DHR96 mutant	0.85	0.89
E-GEOD-24978	3	3	wild type genotype	rbf120a	0.93	0.87
E-GEOD-8775	2	2	female|wild type genotype	female|BG00817 mutant	0.74	0.78
E-MTAB-849	3	3	control	dL3MBT	0.74	0.76
E-GEOD-31564	3	3	eater-N RNAi|none|30 minute	eater-N RNAi|mixture of Gram-positive and Gram-negative bacteria|90 minute	0.73	0.75
E-MTAB-849	3	3	control	dLint1	0.72	0.68
E-GEOD-8775	2	2	female|wild type genotype	female|esg mutant	0.66	0.68
E-GEOD-24978	3	3	wild type genotype	rbf120a wtsX1Lats	0.77	0.67
E-GEOD-8775	2	2	female|wild type genotype	female|CG10990 mutant	0.66	0.66
E-GEOD-35439	3	3	wild type genotype	key1	0.68	0.66
E-GEOD-37701	3	3	vehicle	protocatechuic aldehyde 0.1	0.63	0.65

				millimolar		
E-GEOD-8775	2	2	female|wild type genotype	female|mub mutant	0.64	0.65
E-GEOD-8775	2	2	female|wild type genotype	female|CG9238 mutant	0.64	0.65
E-GEOD-31564	3	3	eater-N RNAi|none|30 minute	eater-N RNAi|mixture of Gram-positive and Gram-negative bacteria|30 minute	0.63	0.65
E-GEOD-25267	3	3	GMR-Gal4/+	GMR-Gal4/+; UAS-dE2F1,UAS-dDP/+	0.63	0.61
E-GEOD-37148	3	3	45 day|wild type drosophila SOD1 expressed in motoneurons	45 day|G85R expressed in motoneurons	0.63	0.61
E-GEOD-8775	2	2	male|wild type genotype	male|BG00817 mutant	0.63	0.61
E-GEOD-31564	3	3	pBR322 RNAi|none|30 minute	pBR322 RNAi|mixture of Gram-positive and Gram-negative bacteria|90 minute	0.58	0.60
E-GEOD-26717	3	3	w; sensDF2RES/+; sensE1/+	w; sensDF2RES/sensDF2RES; sensE1/sensE1	0.61	0.60
E-GEOD-10940	3	3	control|abdomen	Logjam mutant|abdomen	0.61	0.60
E-GEOD-31564	3	3	pBR322 RNAi|none|30 minute	pBR322 RNAi|mixture of Gram-positive and Gram-negative bacteria|30 minute	0.58	0.60
E-GEOD-8938	3	3	uninfected|2 to 5 hour	Leptopilina boulardi (strain Lb17)|2 to 5 hour	0.61	0.60
E-GEOD-24978	3	3	wild type genotype	wtsX1Lats	0.72	0.60
E-GEOD-26246	3	3	wild type genotype|2 day	Wild type Atro transgene|2 day	0.59	0.59
E-GEOD-31564	3	3	eater-N RNAi|none|30 minute	eater-N RNAi|mixture of Gram-positive and Gram-negative bacteria|180 minute	0.60	0.59
E-GEOD-25267	3	3	control	GMR-Gal4/UAS-miR-11; UAS-dE2F1,UAS-dDP/+	0.55	0.59
E-GEOD-10940	3	3	control|head/thorax	Logjam mutant|head/thorax	0.64	0.59
E-GEOD-14058	3	3	control	delg613 mutant	0.55	0.59
E-MEXP-2082	3	4	0 g gravitation (0g*)|19 degree Celsius|male|22 day	1 g gravitation control|19 degree Celsius|male|22 day	0.53	0.58

S7 Table Ranking EBI's ExpressionAtlas (starvation-sensitive molecular signature top 30 experiments). FDR p-value is the p-value corrected for multiple hypothesis testing using False Discovery Rate, accounting for all GO terms tested. FDR p-values < 0.05 are labelled in bold; Where there were multiple factor values, these are separated by "|". Factor values comprise genotype, treatment, etc.

EBI Experiment ID	N con	N mut	EBI Control Assay ID	EBI Mutant Assay ID	µ Control Prob	µ Mutant Prob
E-GEOD-48145	3	3	wild type genotype	ovoD mutant	1.00	0.99
E-GEOD-48145	3	3	wild type genotype	CA knockout with ovoD mutant	0.97	0.96
E-GEOD-10940	3	3	control|abdomen	Logjam mutant|abdomen	0.95	0.96
E-GEOD-10940	3	3	control|head/thorax	Logjam mutant|head/thorax	0.92	0.94
E-MTAB-3546	4	4	3 week|normal conditions	3 week|response to cold	0.89	0.91
E-GEOD-8775	2	2	female|wild type genotype	female|mub mutant	0.84	0.87
E-GEOD-8775	2	2	female|wild type genotype	female|esg mutant	0.74	0.85
E-GEOD-55187	3	4	wild type genotype|female	Sesb1 mutation|female	0.90	0.85
E-GEOD-26726	3	3	10 day|normal|wild type genotype|Canton-S	10 day|restricted|wild type genotype|Canton-S	0.88	0.84
E-GEOD-26726	3	3	40 day|normal|wild type genotype|Canton-S	40 day|restricted|wild type genotype|Canton-S	0.80	0.79
E-GEOD-12834	4	4	unmated	double mated	0.77	0.75
E-GEOD-55187	3	4	wild type genotype|male	Sesb1 mutation|male	0.76	0.71
E-MTAB-1066	3	3	wild type genotype	cycC mutant	0.68	0.70
E-GEOD-12834	4	4	unmated	single mated	0.71	0.70
E-GEOD-14531	3	3	normal|EP2449(precise excision)/KG08976(precise excision)	starvation|EP2449(precise excision)/KG08976(precise excision)	0.64	0.70
E-MEXP-2082	3	3	1 g gravitation control|14 degree Celsius|female|26 hour	1 g gravitation (1g*)|14 degree Celsius|female|26 hour	0.72	0.69
E-TABM-297	3	3	wild type genotype	24BGal4/UAS-lbe	0.64	0.69
E-MEXP-1208	3	3	wild type genotype	Ada2a delta 189	0.70	0.67
E-GEOD-30362	3	3	wild type genotype	pex1 homozygous mutant	0.64	0.67
E-GEOD-48997	5	5	wild type genotype	pri -/- mutant	0.66	0.67
E-GEOD-8938	3	3	uninfected|2 to 5 hour	Leptopilina boulardi (strain Lb17)|2 to 5 hour	0.60	0.66
E-MTAB-1066	3	3	wild type genotype	cdk8 mutant	0.62	0.66
E-GEOD-26726	3	3	10 day|normal|sir2 control|yw, w1118	10 day|normal|sir2 overexpression|yw, w1118	0.73	0.66
E-GEOD-8775	2	2	male|wild type genotype	male|mub mutant	0.45	0.65
E-GEOD-31875	4	4	control	elav-GAL4; UAS-DsRed-CAG100	0.57	0.65

				(5x)		
E-GEOD-44090	3	3	tub-Gal4	sage overexpressed tub-Gal4	0.56	0.65
E-MEXP-1208	3	3	wild type genotype	Gcn5[E333st] / Gcn5[E333st]	0.61	0.65
E-GEOD-25988	3	3	wild type genotype	BxJ mutant	0.61	0.65
E-MEXP-2011	3	3	wild type genotype	Nurf301-A/Nurf301-B/Nurf301-C knockout	0.57	0.65
E-GEOD-31564	3	3	eater-N RNAi|none|30 minute	eater-N RNAi|mixture of Gram-positive and Gram-negative bacteria|90 minute	0.65	0.64

S8 Table Ranking EBI's ExpressionAtlas (sterile molecular signature top 30 experiments). FDR p-value is the p-value corrected for multiple hypothesis testing using False Discovery Rate, accounting for all GO terms tested. FDR p-values < 0.05 are labelled in bold; Where there were multiple factor values, these are separated by "|". Factor values comprise genotype, treatment, etc.

References

1. Gautier L, Cope L, Bolstad BM, Irizarry RA. affy--analysis of Affymetrix GeneChip data at the probe level. Bioinformatics. 2004;20(3):307-15. doi: 10.1093/bioinformatics/btg405. PubMed PMID: 14960456.

2. Breslin T, Eden P, Krogh M. Comparing functional annotation analyses with Catmap. BMC Bioinformatics. 2004;5:193. Epub 2004/12/14. doi: 10.1186/1471-2105-5-193. PubMed PMID: 15588298; PubMed Central PMCID: PMCPMC543458.

3. Sieber MH, Thummel CS. The DHR96 nuclear receptor controls triacylglycerol homeostasis in Drosophila. Cell Metab. 2009;10(6):481-90. Epub 2009/12/01. doi: 10.1016/j.cmet.2009.10.010. PubMed PMID: 19945405; PubMed Central PMCID: PMCPMC2803078.

4. Varghese J, Lim SF, Cohen SM. Drosophila miR-14 regulates insulin production and metabolism through its target, sugarbabe. Genes Dev. 2010;24(24):2748-53. Epub 2010/12/17. doi: 10.1101/gad.1995910. PubMed PMID: 21159815; PubMed Central PMCID: PMCPMC3003191.

5. Nicolay BN, Bayarmagnai B, Islam AB, Lopez-Bigas N, Frolov MV. Cooperation between dE2F1 and Yki/Sd defines a distinct transcriptional program necessary to bypass cell cycle exit. Genes Dev. 2011;25(4):323-35. Epub 2011/02/18. doi: 10.1101/gad.1999211. PubMed PMID: 21325133; PubMed Central PMCID: PMCPMC3042156.

6. van Bergeijk P, Heimiller J, Uyetake L, Su TT. Genome-wide expression analysis identifies a modulator of ionizing radiation-induced p53-independent apoptosis in Drosophila melanogaster. PLoS One. 2012;7(5):e36539. Epub 2012/06/06. doi: 10.1371/journal.pone.0036539. PubMed PMID: 22666323; PubMed Central PMCID: PMCPMC3362589.

7. Magwire MM, Yamamoto A, Carbone MA, Roshina NV, Symonenko AV, Pasyukova EG, et al. Quantitative and molecular genetic analyses of mutations increasing Drosophila life span. PLoS Genet. 2010;6(7):e1001037. Epub 2010/08/06. doi: 10.1371/journal.pgen.1001037. PubMed PMID: 20686706; PubMed Central PMCID: PMCPMC2912381.

8. Boltz KA, Carney GE. Loss of p24 function in Drosophila melanogaster causes a stress response and increased levels of NF-kappaB-regulated gene products. BMC Genomics. 2008;9:212. Epub 2008/05/10. doi: 10.1186/1471-2164-9-212. PubMed PMID: 18466616; PubMed Central PMCID: PMCPMC2396179.

9. Yamamoto R, Bai H, Dolezal AG, Amdam G, Tatar M. Juvenile hormone regulation of Drosophila aging. BMC Biol. 2013;11:85. Epub 2013/07/20. doi: 10.1186/1741-7007-11-85. PubMed PMID: 23866071; PubMed Central PMCID: PMCPMC3726347.

10. Tootle TL, Williams D, Hubb A, Frederick R, Spradling A. Drosophila eggshell production: identification of new genes and coordination by Pxt. PLoS One. 2011;6(5):e19943. Epub 2011/06/04. doi: 10.1371/journal.pone.0019943. PubMed PMID: 21637834; PubMed Central PMCID: PMCPMC3102670.

11. Soshnev AA, Baxley RM, Manak JR, Tan K, Geyer PK. The insulator protein Suppressor of Hairy-wing is an essential transcriptional repressor in the Drosophila ovary. Development. 2013;140(17):3613-23. Epub 2013/07/26. doi: 10.1242/dev.094953. PubMed PMID: 23884443; PubMed Central PMCID: PMCPMC3742144.

12. Peters NC, Thayer NH, Kerr SA, Tompa M, Berg CA. Following the 'tracks': Tramtrack69 regulates epithelial tube expansion in the Drosophila ovary through Paxillin, Dynamin, and the homeobox protein Mirror. Dev Biol. 2013;378(2):154-69. Epub 2013/04/03. doi: 10.1016/j.ydbio.2013.03.017. PubMed PMID: 23545328; PubMed Central PMCID: PMCPMC4141043.

13. Zamparini AL, Davis MY, Malone CD, Vieira E, Zavadil J, Sachidanandam R, et al. Vreteno, a gonad-specific protein, is essential for germline development and primary piRNA biogenesis in Drosophila. Development. 2011;138(18):4039-50. Epub 2011/08/13. doi: 10.1242/dev.069187. PubMed PMID: 21831924; PubMed Central PMCID: PMCPMC3160098.
